# Supplementary material for: The Role of Fatty Acid-Binding Protein 4 in the Characterization of Atrial Fibrillation and the Prediction of Outcomes after Catheter Ablation
Source: Int J Mol Sci. 2022 Sep 21;23(19):11107. doi: 10.3390/ijms231911107 (PMC9570077; doi:10.3390/ijms231911107)
Supplement: Supplementary file 1 [file ijms-23-11107-s001.zip › ijms-1897306-supplementary.pdf]

Supplementary Table S1. Clinical characteristics of patients by gender.

	Women (n=98)	Men (n=201)	*Sig. (p-value)*
**Age**	63±8	56±11	**<0.001**
**BMI (Kg/m2)**	30±5	30±4	0.893
**AF type (1/2/3), (%)**	45%/34%/21%	27%/48%/25%	**0.007**
**LA LVA (%)**	4.3 (0.4-15)	1 (0.1-5.6)	**0.001**
**Years (AF)**	3 (1-6)	2 (1-5)	0.173
**QRS (ms)**	85.5 (80-95)	90 (80-100)	**0.007**
**LA area (cm2)**	19±7	21±6	**0.023**
**LVEF**	61±7	60±9	0.334
**Laboratory measurements**			
Hemoglobin (g/dL)	13.3±1.3	14.7±1.2	**<0.001**
Platelets (10^9/L)	210±39	199±52	**0.027**
Blood urea nitrogen (mg/dL)	46±16	46±13	0.879
Creatinine (mg/dL)	0.9±0.2	1±0.2	**<0.001**
eGFR (mL/ min/ 1.73 m2)	84±27	101±32	**<0.001**
Plasma Sodium (mEq/L)	141±2	141±9	0.984
Plasma Potassium (mEq/L)	4.2 (4-4.45)	4.2 (4-4.4)	0.442
Total cholesterol (mg/dL)	191 (165-220)	188 (158-213)	0.241
LDLc (mg/dL)	112 (89-136)	116 (93-137.5)	0.652
HDLc (mg/dL)	58 (48-67)	46 (40-54)	**<0.001**
Triglycerides (mg/dL)	94 (80-117)	111 (85-147)	**0.004**
Glucose (mg/dL)	104 (91-113)	101 (94-114)	0.919
HbA1c (g/dL)	5.8±0.7	5.7±0.5	0.070
TSH (mU/L)	2.61 (1.67-3.6)	2.17 (1.46-3.19)	0.076
LA Gal-3 (ng/mL)	8.5 (6.9-13.6)	8.6 (5.5-12.7)	0.280
Peripheral Gal-3 (ng/mL)	8.6 (6.2-14.2)	8.5 (5.5-13.5)	0.555
LA FABP4 (ng/mL)	21.7 (14.9-29.8)	12.8 (8.5-20.8)	**<0.001**
Peripheral FABP4 (ng/mL)	25.7 (18.8-39.8)	16.1 (10.7-25.7)	**<0.001**
LA Leptin (ng/mL)	24.8 (13.9-45)	8.4 (4.8-16)	**<0.001**
Peripheral Leptin (ng/mL)	30 (18.9-56.2)	11.4 (6.5-21.9)	**<0.001**
**Disease and risk factors**			
AHT (no/yes)	54%/46%	55%/45%	0.799
T2DM (no/yes)	89%/11%	88%/12%	0.808
Smoker (no/yes)	84.5%/15.5%	65.5%/34.5%	**0.002**
COPD (no/yes)	94%/6%	94%/6%	0.917
OSA (no/yes)	100%/0%	92%/8%	**0.003**
**Treatments**			
Statins (no/yes)	56%/44%	57%/43%	0.810
ACEi (no/yes)	90%/10%	77%/23%	**0.008**
ARB (no/yes)	73%/27%	78%/22%	0.326

Vitamin K antagonist (no/yes)	54%/46%	69%/31%	**0.006**
DOAC (no/yes)	46%/54%	31%/69%	**0.012**
Class I ADT (no/yes)	65%/35%	70%/30%	0.384
Class II ADT (no/yes)	32%/68%	30%/70%	0.737
Class III ADT (no/yes)	70%/30%	72%/28%	0.745
Class IV ADT (no/yes)	91%/9%	94%/6%	0.372

BMI: Body Mass Index; **AF type:** Atrial Fibrillation type (1: paroxysmal; 2: persistent; 3: long-standing persistent); **LA:** Left atrium; **LVA:** Low-voltage areas; **LVEF:** Left Ventricular Ejection Fraction; **eGFR:** Estimated Glomerular Filtration Rate; **Gal-3:** Galecin-3; **FABP4:** Fatty Ac-id-Binding Protein 4; **AHT:** Arterial Hypertension; **T2DM:** Type 2 Diabetes Mellitus; **COPD:** Chronic Obstructive Pulmonary Disease; **OSA:** Obstructive Sleep Apnea; **ACEi:** Angioten-sin-Converting Enzyme inhibitors; **ARB:** Angiotensin Receptor Blockers; **DOAC:** Direct Oral Anticoagulants; **ADT:** Antiarrhyth-mic Drug Therapy.
